# miR-125a suppresses viability and glycolysis and induces apoptosis by targeting Hexokinase 2 in laryngeal squamous cell carcinoma

**DOI:** 10.1186/s13578-017-0178-y

**Published:** 2017-10-05

**Authors:** Zhanwei Sun, Wenqi Zhang, Qian Li

**Affiliations:** 1 Department of Otolaryngology, Henan Provincial People’s Hospital, People’s Hospital of Zhengzhou University, Zhengzhou, 450000 China; 2 Department of Otolaryngology, Henan Provincial People’s Hospital, People’s Hospital of Zhengzhou University, No. 7 Weiwu Road, Zhengzhou, 450003 People’s Republic of China

**Keywords:** miR-125a, Proliferation, Glycolysis, Apoptosis, HK2

## Abstract

**Background:**

miR-125a usually functions as a tumor suppressor in cancers. However, the role of miR-125a in laryngeal squamous cell carcinoma (LSCC) has not been determined.

**Methods:**

qRT-PCR was applied to measure the expression of miR-125a and HK2 mRNA in LSCC tissues and cells. CCK-8 kit and flow cytometry analysis were performed to detect cell viability and apoptosis. Luciferase reporter assay and RNA immunoprecipitation (RIP) were conducted to confirm the relationship between miR-125a and HK2. Commercial test kits were used to determine the concentrations of glucose and l-lactate. Xenograft in mice was constructed to validate the function and mechanism of miR-125a in LSCC tumor growth.

**Results:**

A negative correlation was found between miR-125a expression and the level of Hexokinase 2 (HK2) mRNA in LSCC tissues. Functional experiments found that miR-125a inhibited viability and glycolysis and induced apoptosis in LSCC cells. Similarly, HK2 downregulation led to viability and glycolysis inhibition and induction of apoptosis in LSCC cells in vitro. Moreover, miR-125a overexpression suppressed LSCC xenograft growth in vivo. Mechanically, HK2 was verified to be a target of miR-125a by luciferase reporter assays and RNA immunoprecipitation (RIP) assays. Furthermore, restored HK2 expression reversed miR-125a-mediated proliferation and glycolysis inhibition and induction of apoptosis in LSCC cells.

**Conclusions:**

miR-125a suppressed LSCC progression by targeting HK2 in vitro and in vivo, suggesting that miR-125a may be a potential molecular target for LSCC treatment.

**Electronic supplementary material:**

The online version of this article (doi:10.1186/s13578-017-0178-y) contains supplementary material, which is available to authorized users.

## Background

Laryngeal squamous cell carcinoma (LSCC) is one of head and neck squamous cell carcinomas, with a highly aggressive malignancy [1]. About 2.4% of the world new malignancy cases are LSCC, of which the incidence and mortality rates are rapidly increasing worldwide [2, 3]. Despite great advances achieved in LSCC diagnosis and treatment, patients with LSCC display a low cure rate and high morbidity [4]. Therefore, to completely understand the underlying molecular mechanisms of laryngeal carcinogenesis and to identify novel effective molecules for therapy of LSCC are crucial.

MicroRNAs (miRNAs) are a class of small endogenously non-coding RNA molecules with approximately 22 nucleotides in length, which negatively regulate their target genes by binding to mRNA targets, leading to mRNA degradation or translational repression [5]. Mounting studies have evidenced that many miRNAs function as oncogenes or tumor suppressor in LSCC. For instance, miR-16, acting as an oncogene, led to the inhibition of the cell adhesion capability and the promotion of cell migration in LSCC through directly suppressing Zyxin expression [6]. In addition, Xu et al. demonstrated that miR-106b could increase the proliferation and invasion of laryngeal carcinoma cells through targeting RUNX3, a critical tumor suppressor in many human cancer types [7]. Oppositely, miR-24, functioning as a tumor suppressor in LSCC, could significantly suppress cell proliferation and invasion ability of Hep2 cells via downregulation of S100A8 [8]. Moreover, miR-203 was downregulated in the LSCC tissues, and restored miR-203 expression suppressed cellular proliferation, invasion and induced apoptosis, G1 phase cell cycle arrest of Hep-2 cells through targeting ASAP1 [9]. All these studies demonstrate that miRNAs play significant roles in LSCC. miR-125a is usually low-expressed in many cancers, such as medulloblastoma, gastric cancer, and breast cancer, promoting the cancer progression [10–12]. miRNA expression profiles between laryngeal carcinoma tissue and adjacent normal tissue specimens by both microarray and qRT-PCR analyses indicated that miR-125a was downregulated [13]. Moreover, Liu et al. [14] found that miR-125a was decreased in laryngeal carcinoma tissues and Hep-2 laryngeal cancer stem cells (Hep-2-CSCs) and re-expressing miR-125a enhanced the the sensitivity of Hep-2-CSCs to cisplatin in laryngeal cancer stem cells by targeting HAX-1. However, the function and molecular mechanisms of miR-125a in LSCC progression control have not been determined.

Cancer cells produce energy preferentially by aerobic glycolysis rather than oxidative phosphorylation, which is well known as the Warburg effect [15]. The first and irreversible step of aerobic glucose metabolism is phosphorylating glucose to glucose-6-phosphate, which is catalyzed by hexokinases [16]. Hexokinase 2 (HK2), the major isozyme contributing to aerobic glycolysis, is overexpressed in cancers and proposed as a metabolic target for cancer therapeutic development [17, 18]. Emerging documents have demonstrated that increased HK2 expression leads to promotion of cell proliferation and inhibition of apoptosis in many cancers including ovarian, breast and lung cancer [19–21]. Moreover, HK2 overexpression promotes the proliferation, aerobic glycolysis and inhibits apoptosis in LSCC [22, 23]. However, how HK2 is regulated is not well elucidated in LSCC.

In the present study, we aimed to investigate the role and potential molecular mechanisms of miR-125a in LSCC progression.

## Methods

### Tissue specimens and cell culture

Twenty-three LSCC tissue samples were obtained from patients in the People’s Hospital of Zhengzhou University. Among the 23 tissues, 11 were Stage 1, 2 tumor tissues and 12 were Stage 3, 4 tumor tissues. This study was approved by the Ethic Review Committees of the People’s Hospital of Zhengzhou University. Written consents were obtained from all patients.

Two human LSCC cell lines (AMC-HN-8 and TU212) obtained from the American Type Culture Collection (ATCC, Manassas, VA, USA) were cultured at 37 °C in a humidified 5% CO_2_ incubator. All cells were grown in DMEM (Invitrogen, Carlsbad, CA, USA) supplemented with 10% fetal bovine serum and 1% penicillin/streptomycin.

### Quantitative real-time PCR (qRT-PCR)

Total RNA was extracted from AMC-HN-8 and TU212 cells or LSCC patient tissues using TRIzol (Invitrogen) and cDNA was generated by ImProm-II reverse transcription system (Promega, Madison, WI, USA). qRT-PCR was performed using SYBR Premix Ex Taq™ (Takara Bio, Shiga, Japan) on the 7500 Real Time PCR System (Applied Biosystems, Foster City, CA, USA). The qPCR results were analyzed using the 2^−ΔΔCt^ method.

### Cell transfection

Si-HK2 (5′-CCGTAACATTCTCATCGATTT-3′), Si-control, miR-125a mimic, miR-control (scramble miRNA), miR-125a inhibitor (anti-miR-125a) and inhibitor control anti-miR-control (scramble miRNA) were purchased from GenePharma (Shanghai, China). HK2-overexpressing plasmid pcDNA-HK2 was constructed by amplifying HK2 from the cDNA of AMC-HN-8 cells and cloning into the pcDNA3.1 vector. Cells transfection was performed using Lipofectamine 2000 (Invitrogen).

### Cell viability assay

Cell viability was measured by using the Cell Counting Kit-8 (CCK-8; Dojindo, Kumamato, Japan) according to the manufacturer’s protocol. Briefly, AMC-HN-8 and TU212 cells (2 × 10^3^ per well) were seeded in a 96-well plate. After cell transfection for 48 h, CCK-8 reagent (10 µL) was added to the cells for 2 h. Then, the optical density (OD) of 450 nm was determined by a microplate reader (Bio-Rad Labs, Sunnyvale, CA, USA).

### Apoptosis assay

Apoptosis was measured using the Annexin V apoptosis detection kit (BD bioscience, San Jose, CA, USA). AMC-HN-8 and TU212 cells were collected and incubated with FITC-Annexin V and propidium iodide at 48 h after transfection. Then, flow cytometry was performed on the FACScan flow cytometer (Becton–Dickinson, Mountain View, CA, USA).

### Glucose consumption and l-lactate production

Glucose consumption and lactate production were analyzed as described previously [24]. The concentrations of glucose and l-lactate were determined using a glucose test kit (Applygen Technologies, Beijing, China) and a l-lactate assay kit (Bioassay Systems, Hayward, CA, USA), respectively.

### Western blot

Total protein was extracted by using RIPA reagents (Thermo Scientific, Rockford, IL, USA) and the protein level was detected using the Bradford Protein Assay Kit (Beyotime, Haimen, China). Then, proteins were separated by SDS-PAGE, transferred to a nitrocellulose membrane, and detected using specific antibodies. The following antibodies were used: anti-HK2 (Santa Cruz Biotechnology Inc., Santa Cruz, CA, USA), anti-Ki-67, anti-Caspase-3 and anti-β-actin (Sigma, St Louis, MO, USA). The protein bands were visualized by using the ECL chemiluminescent reagent kit (Amersham Biosciences, Buckinghamshire, UK).

### Luciferase reporter assays

For HK2 3′ UTR luciferase reporter assay, the wild or mutant type reporter vectors (HK2-WT or HK2-MUT) were co-transfected into AMC-HN-8 and TU212 cells together with miR-125a mimics or miR-control. The cells were harvested at 48 h after transfection. The luciferase activity was measured with Dual-Luciferase reporter assay system (Promega).

### RNA immunoprecipitation (RIP) assay

RNA immunoprecipitation was performed using the Magna RIP Kit (Millipore, Billerica, MA, USA) according to the manufacturer’s instruction. Briefly, AMC-HN-8 and TU212 cells were lysed in RIP lysis buffer, then 100 μl of whole cell extract was incubated with RIP buffer containing magnetic beads conjugated with human anti-Ago2 antibody, positive control anti-snRNP70 or negative control normal mouse IgG (Millipore). To digest the protein, samples were incubated with Proteinase K with shaking. Then immunoprecipitated RNA was isolated. Finally, the levels of miR-125a and HK2 mRNA in the precipitates were detected by qRT-PCR.

### Xenograft in nude mice

For miR-125a functional study in vivo, miR-125a- or miR-control-overexpressing AMC-HN-8 cells were subcutaneously injected into the right flank of the male athymic BALB/c nude mice (n = 6 per group). Tumor volume was monitored every 3 days, and mice were killed on day 21 for tumor weight analysis. Then, the mRNA and protein levels of HK2 in subcutaneous tumors were detected by qRT-PCR and western blot analysis. Additionally, the protein levels of Ki-67 and Caspase-3 were also determined by western blot analysis. All animal procedures were performed with the approval of the Medical Experimental Animal Care Commission of Zhengzhou University.

### Statistical analysis

Data were analyzed using SPSS 19.0 statistics software (SPSS, Chicago, IL, USA) and presented as mean ± SD. Student’s *t* test and one-way ANOVA were used to evaluate the significance. Differences were considered to be significant when *P* < 0.05.

## Results

### miR-125a expression is negatively correlated with the level of HK in LSCC tissues

**Fig. 1:**
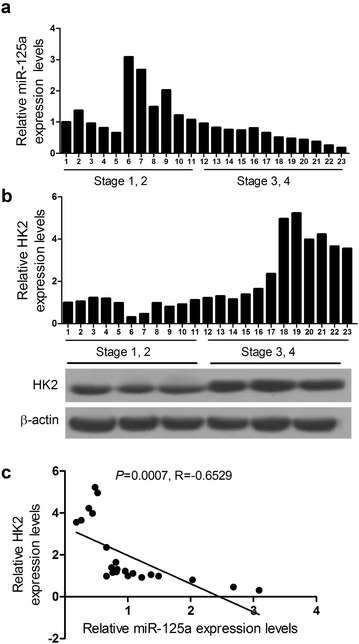
A negative correlation between miR-125a and HK2 mRNA exists in LSCC. Total RNA of LSCC tissues form 23 patients (11 Stage 1, 2 and 12 Stage 3, 4) was extracted and reverse-transcripted into cDNA, then qRT-PCR analysis was performed. **a** The relative expression levels of miR-125a in LSCC tissues. **b** The relative HK2 mRNA and protein expression levels in LSCC tissues. **c** miR-125a expression is negatively correlated with the level of HK2 in LSCC tissues

To detect the expression level of miR-125a and HK2 mRNA in LSCC tissues, total RNA of LSCC tissues form 23 patients (11 Stage 1, 2 and 12 Stage 3, 4) was extracted and reverse-transcripted into cDNA, then qRT-PCR analysis was performed. qRT-PCR analysis revealed that miR-125a expression was high in early-stage patients (Stage 1, 2) and low in late-stage patients (Stage 3, 4) (Fig. 1a). The level of HK2 mRNA and protein was low in early-stage patients and high in late-stage patients (Fig. 1b). In addition, miR-125a expression is negatively correlated with the level of HK2 mRNA in LSCC tissues (Fig. 1c). Furthermore, the association between miR-125a and clinical parameters of patients with LSCC was displayed in Table 1. The results showed that high expression of miR-125a was significantly associated with histological grade, tumor stage and lymph node metastasis (*P* < 0.05). All these results suggested that low-expressed miR-125a and elevated HK2 may be involved in LSCC pathogenesis.

**Table 1:** Correlation between miR-125a expression and clinicopathological features of patients with LSCC

Characteristics	Group	Total (n = 23)	miR-125a expression		*P* value
			High (n = 12)	Low (n = 11)	
Gender	Male	13	6	7	0.855
	Female	10	5	5	
Age (years)	< 60	12	6	6	0.827
	≥ 60	11	6	5	
Histological grade	Low	11	9	2	0.006*
	High	12	3	9	
Tumor stage (T)	T1, T2	14	10	4	0.021*
	T3, T4	9	2	7	
Lymph nodes metastasis	No	9	7	2	0.021*
	Yes	14	5	9	

* *P* < 0.05 was considered significantly significant

### miR-125a overexpression inhibits viability and glycolysis and induces apoptosis in LSCC cells

**Fig. 2:**
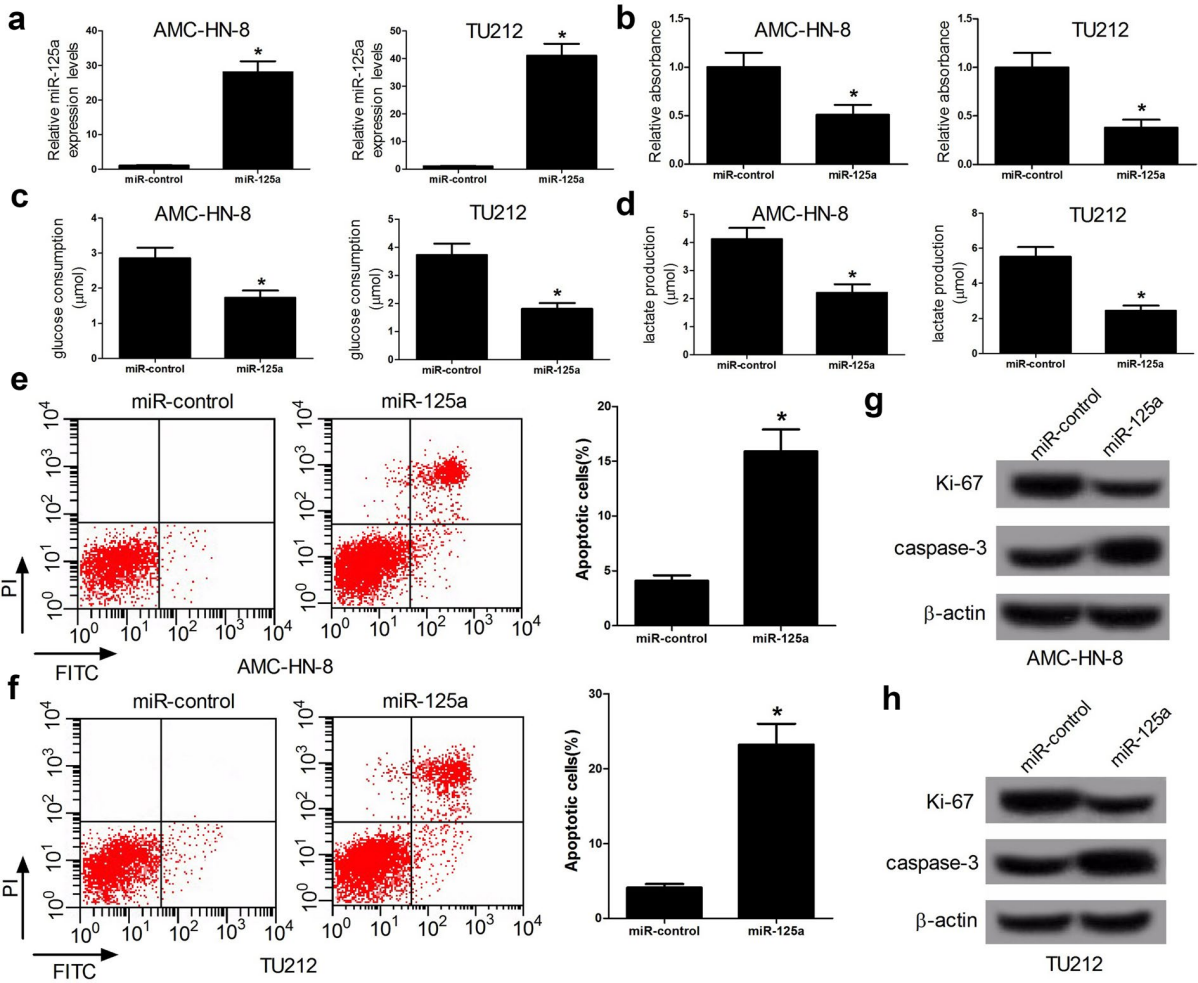
miR-125a overexpression suppresses viability and glycolysis and promotes apoptosis in LSCC cells. AMC-HN-8 and TU212 cells were transfected with miR-125a or miR-control. **a** qRT-PCR analysis was performed to detect the expression of miR-125a in AMC-HN-8 and TU212 cells transfected with miR-125a mimics. **b** The cell viability of AMC-HN-8 and TU212 cells was determined by CCK-8 assays. **c**, **d** Glucose consumption and lactate production in AMC-HN-8 and TU212 cells. **e**, **f** The cell apoptosis of AMC-HN-8 and TU212 cells was determined by flow cytometry analysis. **g**, **h** The protein levels of Ki-67 and Caspase-3 was detected by western blot analysis. *P < 0.05

To demonstrate the functional role of miR-125a in LSCC, the effects of miR-125a overexpression on the viability of AMC-HN-8 and TU212 cells was estimated. As shown in Fig. 2a, miR-125a-overexpressing AMC-HN-8 and TU212 cells were successfully established by transfection with miR-125a mimics. The cell viability of AMC-HN-8 and TU212 cells was significantly suppressed by overexpression of miR-125a when compared with miR-control (Fig. 2b). Given that some miRNAs could regulate glycometabolism, we asked whether miR-125a could also modulate glucose metabolism. To validate the role of miR-125a in glycolysis, the effect of miR-125a on glucose consumption and lactate production was evaluated. The results showed that overexpressed miR-125a remarkedly decreased glucose consumption and lactate production in AMC-HN-8 and TU212 cells (Fig. 2c, d). Then, the effect of miR-125a on apoptosis was further investigated. Flow cytometry analysis confirmed that elevated miR-125a expression led to induction of cell apoptosis in AMC-HN-8 and TU212 cells (Fig. 2e, f). Western bolt analysis also confirmed that miR-125a overexpression inhibited cell viability and enhanced cell apoptosis, evidenced by decreased Ki-67 and elevated Caspase-3 protein levels (Fig. 2g, h). To further investigate the function of miR-125a inhibition in LSCC cells, loss of function assays were performed by transecting anti-miR-con or anti-miR-125a into AMC-HN-8 and TU212 cells. As expected, miR-125a downregulation promoted cell viability and glycolysis and suppressed cell apoptosis in AMC-HN-8 and TU212 cells (Additional file 1: Figure S1A–H). Taken together, these data illuminated that miR-125a suppressed viability and glycolysis and induced apoptosis in LSCC cells.

### HK2 down-regulation suppresses viability and glycolysis and induces apoptosis in LSCC cells

**Fig. 3:**
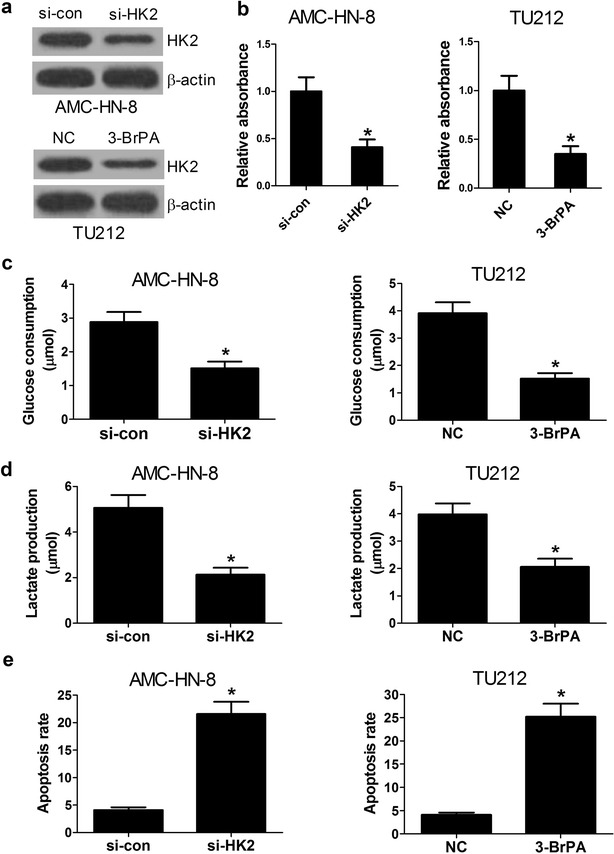
Down-regulation of HK2 inhibits viability and glycolysis and induces apoptosis in LSCC cells. **a** Western blot analysis was performed to detect the protein level of HK2 in AMC-HN-8 and TU212 cells transfected with si-HK2 or treated with an inhibitor of HK2 (3-BrPA). **b** The cell viability of AMC-HN-8 and TU212 cells was determined by CCK-8 assays. **c**, **d** Glucose consumption and lactate production in AMC-HN-8 and TU212 cells. **e**, The cell apoptosis of AMC-HN-8 and TU212 cells was determined by flow cytometry analysis. *P < 0.05. NC represents negative control

Previous studies demonstrated that HK2 overexpression promoted the proliferation, aerobic glycolysis and inhibited apoptosis in LSCC [22, 23]. To further confirmed the effect of HK2 on viability, glycolysis and apoptosis in LSCC, AMC-HN-8 cells were transfected with si-HK2 and TU212 cells were treated with 3-BrPA, and then the viability, glycolysis and apoptosis were determined. At first, the HK2 protein level in AMC-HN-8 and TU212 cells was successfully down-regulated by si-HK2 and 3-BrPA, an inhibitor of HK2 (Fig. 3a), respectively. CCK-8 assay indicated that HK2 down-regulation resulted in reduction of AMC-HN-8 and TU212 cell viability (Fig. 3b). The glycolysis in AMC-HN-8 and TU212 cells was suppressed by si-HK2 and 3-BrPA, evidenced by decreased glucose consumption and lactate production (Fig. 3c, d). Additionally, HK2 inhibition promoted the apoptosis of AMC-HN-8 and TU212 cells (Fig. 3e). Collectively, down-regulation of HK2 led to suppression of viability and glycolysis, and induction of apoptosis in LSCC cells.

### HK2 is targetedly regulated by miR-125a

**Fig. 4:**
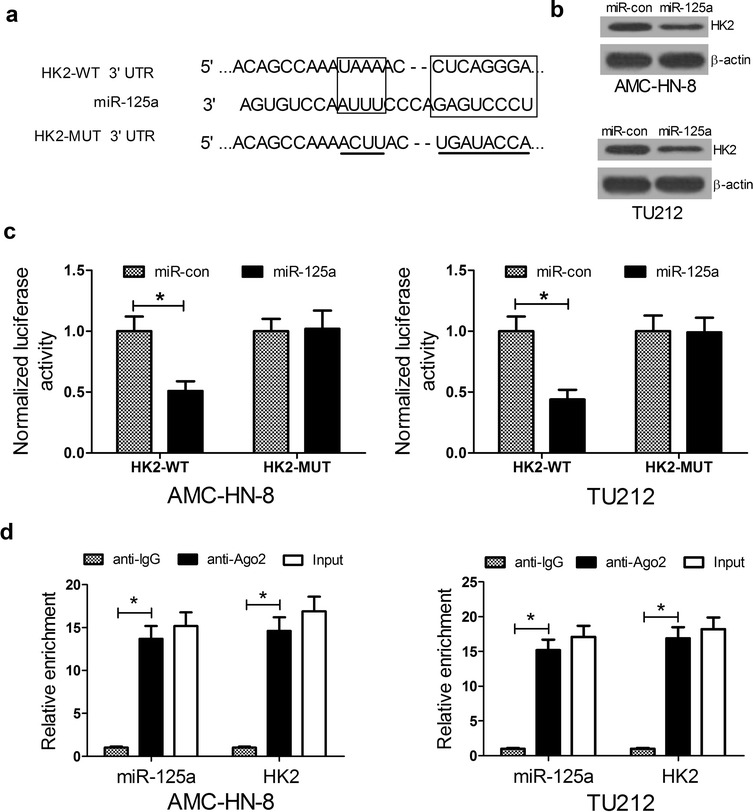
HK2 is a target of miR-125a. **a** The putative binding sites of miR-125a on the 3′ UTR of HK2. **b** The protein levels of HK2 in AMC-HN-8 and TU212 cells 48 h after miR-125a transfection. **c** Relative activity of luciferase reporters with HK2 3′ UTR after co-transfection with miR-125a mimics in AMC-HN-8 and TU212 cells. **d** Cellular lysates from AMC-HN-8 and TU212 cells were used for RNA immunoprecipitation (RIP) with Ago2 antibody. miR-125a and HK2 mRNA were detected using qRT-PCR. *P < 0.05

Considered that the inverse expression level and action of miR-125a and HK2 in LSCC, and that miRNAs usually exerted their function by suppressing their target genes, we further explored the relationship of miR-125a and HK2. As shown in Fig. 4a, HK2 was a predicted target of miR-125a. Western blot analysis revealed that miR-125a overexpression inhibited HK2 protein levels in AMC-HN-8 and TU212 cells. Luciferase reporter assays indicated that the wild-type reporter activity in AMC-HN-8 and TU212 cells was dramatically reduced by miR-125a overexpression, whereas the mutant reporter was not affected (Fig. 4c). It is well known that miRNAs may regulate their targets through forming RNA-induced silencing complex (RISC). To further explore whether both miR-125a and HK2 were in the RISC complex, RIP experiments were performed on AMC-HN-8 and TU212 cell extracts using antibodies against Ago2, a key component of the RISC complex. As expected, miR-125a and HK2 were enriched in Ago2 pellets relative to control IgG immunoprecipitates (Fig. 4d), which was consistent with our bioinformatic analysis and luciferase assays. All these data confirmed that HK2 is a target of miR-125a.

### Restored HK2 expression reversed the inhibited viability and glycolysis, and induced apoptosis in LSCC cells caused by miR-125a overexpression

**Fig. 5:**
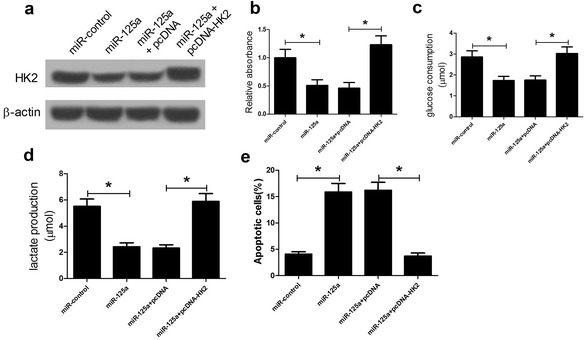
HK2 overexpression reversed the inhibitory effect of miR-125a overexpression on viability, glycolysis, and apoptosis in LSCC cells. **a** The protein levels of HK2 in AMC-HN-8 cells transfected with miR-125a or co-transfected with miR-125a and pcDNA-HK2. **b** The cell viability of AMC-HN-8 cells transfected with miR-125a or co-transfected with miR-125a and pcDNA-HK2. **c**, **d** Glucose consumption and lactate production in AMC-HN-8 cells transfected with miR-125a or co-transfected with miR-125a and pcDNA-HK2. **e** The cell apoptosis of AMC-HN-8 cells transfected with miR-125a or co-transfected with miR-125a and pcDNA-HK2. *P < 0.05

To investigate whether miR-125a regulated viability, glycolysis and apoptosis in LSCC cells through targeting HK2, AMC-HN-8 cells were transfected with miR-125a mimics or co-transfected with miR-125a mimics and pcDNA-HK2. As expected, miR-125a decreased the HK2 protein level, which was abolished by HK2 overexpression (Fig. 5a). The viability of AMC-HN-8 cells was reduced by miR-125a overexpression, which was reversed by pcDNA-HK2 (Fig. 5b). Moreover, HK2 overexpression abolished miR-125a-mediated reduction of glucose consumption and lactate production in AMC-HN-8 cells (Fig. 5c, d). Furthermore, restored HK2 expression reversed the inductive effect of miR-125a on apoptosis in AMC-HN-8 cells (Fig. 5e). All these results demonstrated that miR-125a inhibited viability and glycolysis and induced apoptosis in LSCC cells through targeting HK2.

### miR-125a overexpression suppresses LSCC growth in vivo

**Fig. 6:**
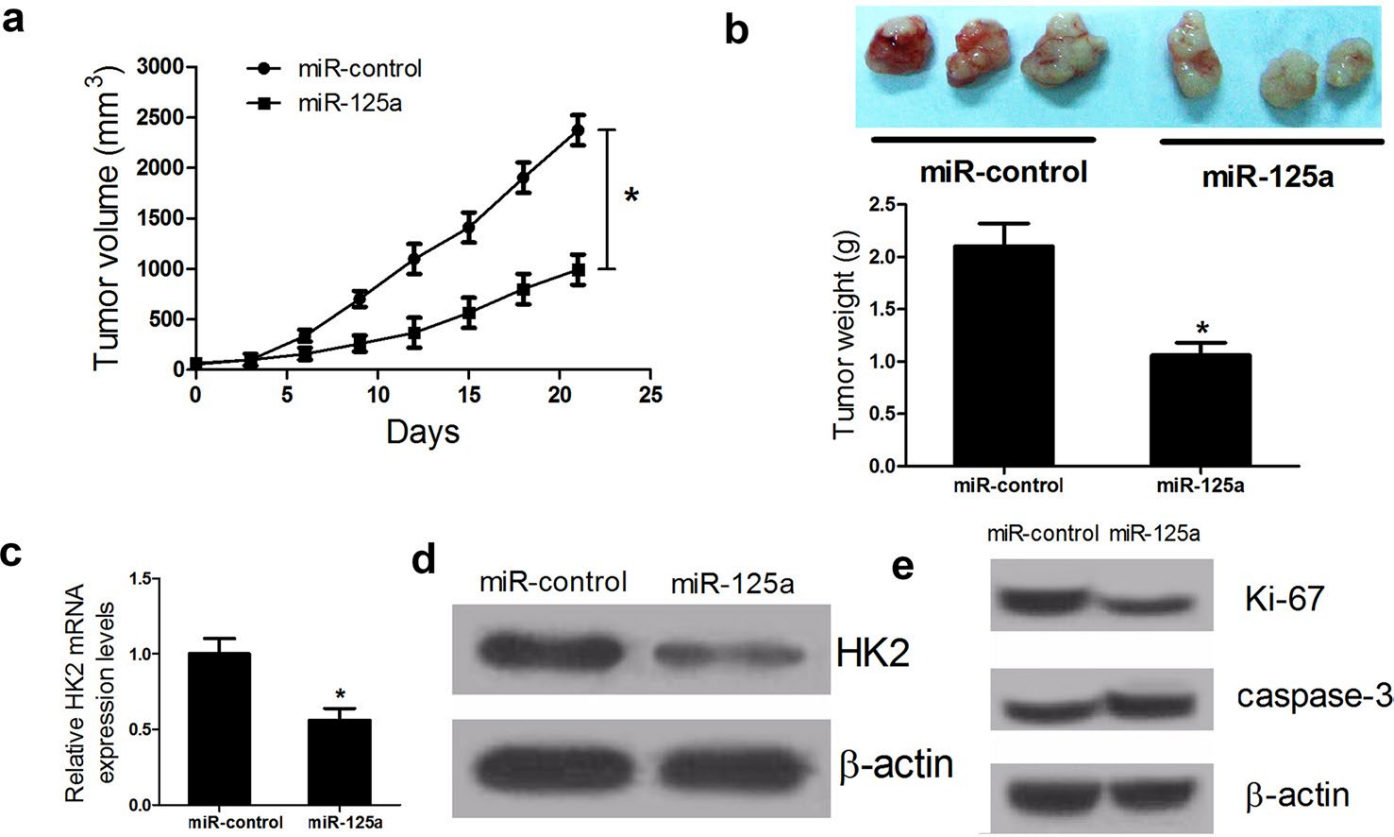
miR-125a overexpression inhibits LSCC xenograft growth in vivo. **a** The tumor volume was calculated every 3 days from day 0 to 21. **b** The average weight of tumors was measured on day 21. **c**, **d** The mRNA and protein levels of HK2 in tissues of resected tumors. **e** The protein levels of Ki-67 and Caspase-3 in subcutaneous tumor tissues. *P < 0.05

The effects of miR-125a overexpression on the in vivo growth were also evaluated. During the whole tumor growth period, tumor volumes from the miR-125a-overexpressing AMC-HN-8 cells were smaller than those of miR-control group (Fig. 6a). After 3-week inoculation, the average weight of tumors developed from the miR-125a-overexpressing AMC-HN-8 cells was lower than that of miR-control group (Fig. 6b). Moreover, miR-125a inhibited HK2 mRNA and protein expression in vivo (Fig. 6c, d). Furthermore, the Ki-67 protein level was reduced and the Caspase-3 protein level was elevated by miR-125a in vivo (Fig. 6e). All these data suggested that miR-125a suppresses LSCC growth in vivo.

## Discussion

Recently, emerging evidence suggests that miRNAs could modulate cellular proliferation, differentiation, migration and invasion, and apoptosis in LSCC. For example, aberrant miR-155 expression promoted Hep-2 cells proliferation, migration and invasion by targeting SOCS1 and elevating STAT3 expression [25]. RARα-mediated miR-27a transcriptional inactivation released the suppression of miR-27a on GSK-3β leading to LSCC differentiation through GSK-3β-involved Wnt/β-catenin pathway [26]. In addition, low-expressed miR-203 was found in LSCC tissues, and functioned as a tumor suppressor through inhibiting proliferation, invasion and inducing apoptosis of Hep-2 cells, which was mediated by ASAP1 [9]. All these documents demonstrated that miRNAs could act as oncogenes or tumor suppressors in LSCC progression.

Previous studies demonstrated that restored miR-125a expression led to the reduction of cell proliferation, migration, and cell death in breast and gastric cancers [27, 28]. Our study found that miR-125a decreased glycolysis in LSCC cells, revealing that miR-125a suppressed glucose metabolism in cancer cells. To our knowledge, this study first demonstrates that this tumor-suppressive miRNA exerts important role in the regulation of cancer cell glycolysis. Moreover, our study also revealed that miR-125a suppressed viability and induced apoptosis in LSCC cells. In agreement with our study, previous documents demonstrated that miR-125a repressed proliferation and promoted apoptosis in other cancers, such as acute myeloid leukemia, colon cancer and renal cell carcinoma [29–31].

HK2, a key glycolytic enzyme for aerobic glycolysis, was identified as a functional target of miR-125a in the present study. Our study also demonstrated that miR-125a repressed glycolysis by targeting HK2 in LSCC. Moreover, HK2 down-regulation inhibited viability and induced apoptosis of LSCC, indicating that HK2 was an oncogene in LSCC. In consistent with our findings, Wolf et al. [32] revealed that HK2 promoted tumor growth in glioblastoma multiforme. HK2 can also associate with mitochondria where they interact with mitochondrial membrane protein voltage-dependent anion channel to control apoptosis process [33]. Furthermore, knockout of HK2 suppressed tumor initiation and progression in KRAS-driven lung cancer and ErbB2-driven breast cancer mouse models [34]. All these studies confirmed that HK2 promoted the progression of cancers. Previous studies suggested that miR-125a functioned as a tumor suppressor through targeting oncogenes such as MMP11 and IL-32Rα [35, 36]. Our findings here revealed that targeting HK2 also contributed to the tumor suppressive activity of miR-125a. These findings illuminated that miR-125a-mediated HK2 inhibition, in addition to regulating glucose metabolism, regulated LSCC tumorigenesis. These findings not only further support the notion that cancer cells use aerobic glycolysis to generate biosynthetic precursors for sustaining cancer cell proliferation [37], but also add a novel molecular link between tumor biology and tumor metabolism.

In addition, due to its role in aerobic glycolysis in cancers, HK2 has been extensively investigated. Epigenetic modification and/or gene amplification was closely related to elevated HK2 expression during tumorigenesis [17]. Additionally, cAMP, insulin, glucose, and oxidative stress also controlled HK2 expression and activity [38]. Our results showed HK2 was targeted by miR-125a. Moreover, HK2 targeted by other miRNAs could modulate the glycolysis in various cancers, such as miR-199a-5p in liver cancer and miR-143 in breast and lung cancers [39–41]. All these studies together with ours demonstrated that miRNAs were also involved in regulation of HK2 expression.

## Conclusions

In summary, this study demonstrated that miR-125a could suppress viability and glycolysis and induce apoptosis in vitro and inhibit tumor growth in vivo in LSCC. Mechanically, the inhibitory effect of miR-125a on LSCC progression was mediated by its target HK2.

## Additional file

**Additional file 1: Figure S1.** miR-125a surpression promotes viability and glycolysis and inhibits apoptosis in LSCCcells. AMC-HN-8 and TU212 cells were transfected with anti-miR-125a or anti-miR-control. *a* qRT-PCRanalysis was performed to detect the expression of miR-125a in AMC-HN-8 and TU212 cells transfectedwith miR-125a mimics. *b* The cell viability of AMC-HN-8 and TU212 cells was determined by CCK-8 assays.*c*, *d* Glucose consumption and lactate production in AMC-HN-8 and TU212 cells. *e*, *f* The cell apoptosis ofAMC-HN-8 and TU212 cells was determined by flow cytometry analysis. *g*, *h* The protein levels of Ki-67 andCaspase-3 was detected by western blot analysis. **P *< 0.05.
