# Supplementary figures and images for: miR-125a suppresses viability and glycolysis and induces apoptosis by targeting Hexokinase 2 in laryngeal squamous cell carcinoma
Source: Cell Biosci. 2017 Oct 5;7:51. doi: 10.1186/s13578-017-0178-y (PMC5629811; doi:10.1186/s13578-017-0178-y)

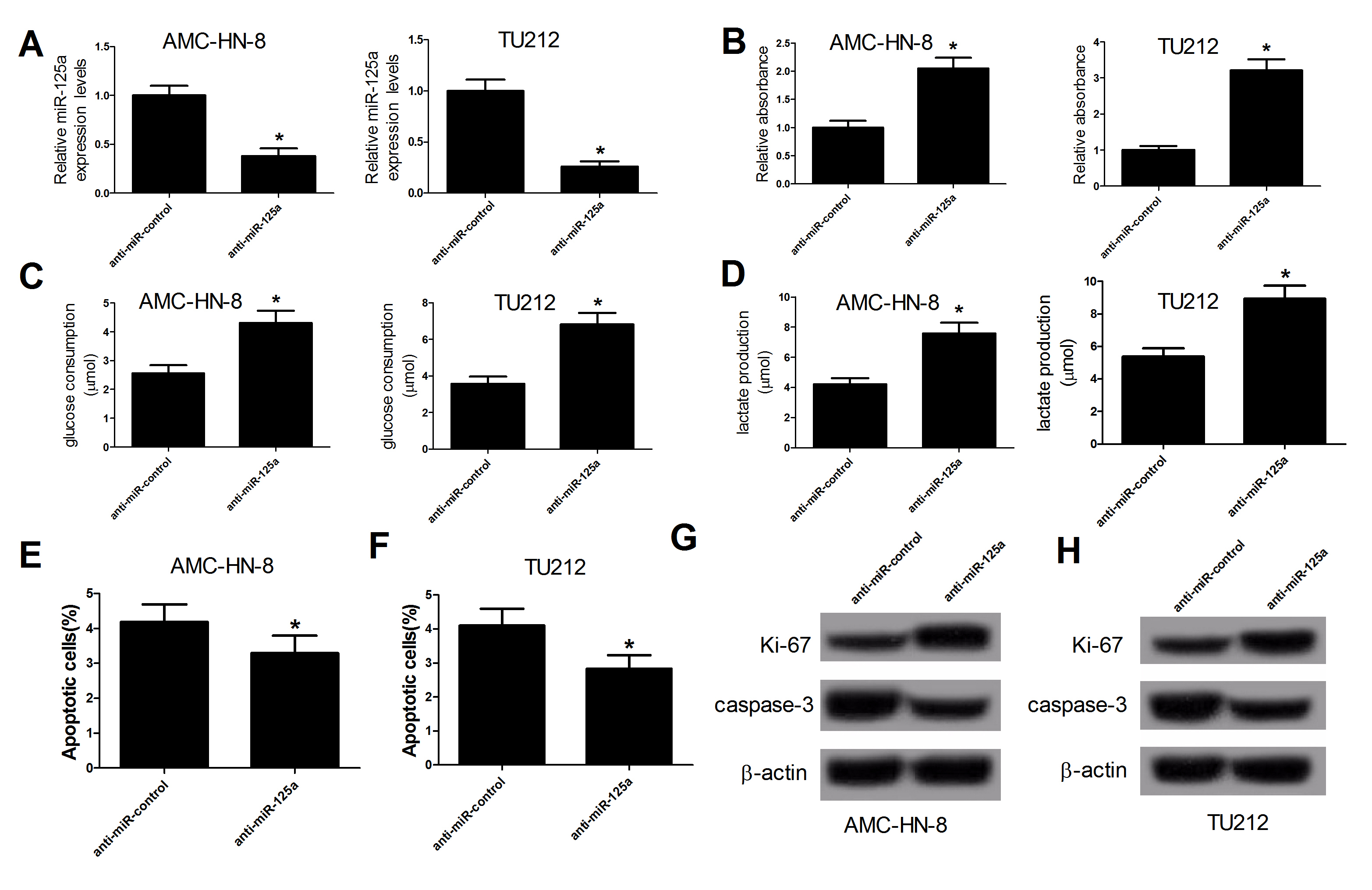

Supplement: Supplementary file 1 — Additional file 1: Figure S1. miR-125a surpression promotes viability and glycolysis and inhibits apoptosis in LSCCcells. AMC-HN-8 and TU212 cells were transfected with anti-miR-125a or anti-miR-control. a qRT-PCRanalysis was performed to detect the expression of miR-125a in AMC-HN-8 and TU212 cells transfectedwith miR-125a mimics. b The cell viability of AMC-HN-8 and TU212 cells was determined by CCK-8 assays.c, d Glucose consumption and lactate production in AMC-HN-8 and TU212 cells. e, f The cell apoptosis ofAMC-HN-8 and TU212 cells was determined by flow cytometry analysis. g, h The protein levels of Ki-67 andCaspase-3 was detected by western blot analysis. *P < 0.05. [file 13578_2017_178_MOESM1_ESM.jpg]
